# Trends and determinants of catastrophic health expenditure in China 2010–2018: a national panel data analysis

**DOI:** 10.1186/s12913-021-06533-x

**Published:** 2021-05-29

**Authors:** Cai Liu, Zhao-min Liu, Stephen Nicholas, Jian Wang

**Affiliations:** 1 School of Management, Tianjin University of Traditional Chinese Medicine, 301617 Tianjin, China; 2 Jining Medical University, 669 Xueyuan Road, Donggang District, 276826 Rizhao City, Shandong Province China; 3 Australian National Institute of Management and Commerce, 1 Central Avenue Australian Technology Park, Eveleigh, NSW 2015 Sydney, Australia; 4 School of Economics and School of Management, Tianjin Normal University, West Bin Shui Avenue, 300074 Tianjin, China; 5 Research Institute for International Strategies, Guangdong University of Foreign Studies, Baiyun Avenue North, 510420 Guangzhou, China; 6 Newcastle Business School, University of Newcastle, University Drive, 2308 Newcastle, NSW Australia; 7 Dong Fureng Institute of Economic and Social Development, Wuhan University, No.54 Dongsi Lishi Hutong, Dongcheng District, 100010 Beijing, China; 8 Center for Health Economics and Management, School of Economics and Management, Wuhan University, 299 Bayi Road, Wuchang District, 430072 Wuhan, Hubei Province China

**Keywords:** Catastrophic health expenditures, Out-of-pocket expenses, Health insurance, China

## Abstract

**Background:**

Catastrophic health expenditures (CHE) are out-of-pocket payments (OOP) that exceed a predefined percentage or threshold of a household’s resources, usually 40 %, that can push households into poverty in China. We analyzed the trends in the incidence and intensity, and explored the determinants, of CHE, and proposed policy recommendation to address CHE.

**Methods:**

A unique 5-year national urban-rural panel database was constructed from the China Family Panel Studies (CFPS) surveys. CHE incidence was measured by calculating headcount (percentage of households incurring CHE to the total household sample) and intensity was measured by overshoot (degree by which an average out of pocket health expenditure exceeds the threshold of the total sample). A linear probability model was employed to assess the trend in the net effect of the determinants of CHE incidence and a random effect logit model was used to analyse the role of the characteristics of the household head, the household and household health utilization on CHE incidence.

**Results:**

CHE determinants vary across time and geographical location. From 2010 to 2018, the total, urban and rural CHE incidence all showed a decreasing tend, falling from 14.7 to 8.7 % for total households, 12.5–6.6 % in urban and 16.8–10.9 % in rural areas. CHE intensity decreased in rural (24.50–20.51 %) and urban (22.31–19.57 %) areas and for all households (23.61–20.15 %). Inpatient services were the most important determinant of the incidence of CHE. For urban households, the random effect logit model identified household head (age, education, self-rated health); household characteristics (members 65 + years, chronic diseases, family size and income status); and healthcare utilization (inpatient and outpatient usage) as determinants of CHE. For rural areas, the same variables were significant with the addition of household head’s sex and health insurance.

**Conclusions:**

The incidence and intensity of CHE in China displayed a downward trend, but was higher in rural than urban areas. Costs of inpatient service usage should be a key intervention strategy to address CHE. The policy implications include improving the economic level of poor households, reforming health insurance and reinforcing pre-payment hospital insurance methods.

**Supplementary Information:**

The online version contains supplementary material available at 10.1186/s12913-021-06533-x.

## Introduction

Illness-caused poverty and poverty-caused illness challenge the well-being of whole societies. Medical costs, especially catastrophic health expenditures, force households to cut back on other consumption, worsen family quality of life and can plunge families into long-term debt and medical poverty [1]. According to the World Health Organization, catastrophic health expenditures (CHE) are out-of-pocket payments (OOP) for medical expenses, not covered by health insurance, ≥ 40 % of total household expenditure minus food spending [1–4]. Globally, CHE has increased significantly [5, 6] and the causes remain unsolved, especially in low and middle income countries. In 2015, about 150 million people worldwide experienced CHE, pushing an estimated 100 million people into poverty [7]. Global per capita OOP rose from $161.00 in 2010 to $187.00 in 2018, a growth rate of 16.15 % [8]. Over the same period in China, total OOP expenses increased from $96.254 billion to $242.687 billion, growing about 152.13 % [9]. Compared with other countries, China suffered a high CHE incidence, about 13 % compared to a 59 cross-country study where the rate of CHE ranged from 0.01 in the Czech Republic and Slovakia to 10.5 % in Vietnam [10].

Like many other developing countries, the incidence of CHE in China reflects the limited health insurance coverage of OOP expenses [4]. In order to lower CHE and OOP expenses, China piloted a series of healthcare reforms in 2009, including three national basic medical insurance (BMI) programs: urban employee basic medical insurance (UEBMI) covering employed urban residents; the urban resident basic medical insurance (URBMI) designed for the urban unemployed, retired, elderly, students and children; and the new rural cooperative medical scheme (NRCMS) for rural residents. Currently, China’s national BMI covers more than 1.35 billion people, with the insured rate reaching 97 % of the population [11]. However, the financial level and benefit package of each BMI differ significantly. For example, the per-capita fund of UEBMI is US$424.7, but only US$66.2 for URBMI and US$61.2 for NRCMS [12, 13]. The UEBMI benefit package is more generous and comprehensive than URBMI and NRCMS, with URBMI and NRCMS having limited outpatient service coverage. Finally, the benefit packages vary by province and city, where the same BMI provides different OOP expenses, reimbursement rates and coverage depending on city-province. To narrow disparities between the three BMI schemes, China started to integrate URBMI and NRCMS into URRMI (Urban and Rural Residents Medical Insurance) scheme in 2016 [14]. To control the rapidly rising health expenditure, these BMI reforms were coupled with public hospital reform measures, limiting medicine payments and increasing the fee-for-service payment method.

Aiming at relieving the financial burden on people who suffer a critical illness, China also launched catastrophic medical insurance (CMI) in 2012, which was implemented nationwide in 2016 after 3 years of city-based testing. CMI was designed to reimburse patients whose OOP medical expenses exceeding a pre-determined BMI level, where claimants could receive a 50–100 % reimbursement, depending on individual provincial policies [15]. Critical illness in the CMI scheme was determined by the patient’s OOP expenses, which was defined by BMI province-level reimbursement policies, irrespective of the kind of disease.

In the context of China’s complex BMI schemes and health reforms, the determinants of CHE have been a topic of continued research interest in China [16]. Empirical studies of the determinants of CHE broadly identify four main causes: first, the characteristics of the household head, including sex, age, education, marital status and self-rated health; second, features of the household [17], such as region, economic status and family size; third, attributes of the family members [18, 19] including the number of old or young family members and family members with a doctor-diagnosed chronic and special diseases, such as cancer [20], diabetes [21] cardiovascular diseases [22]and hypertension [23]; and fourth, health care service utilization and policy related variables, including inpatient and outpatient hospital care services [5, 16], access to health consultation services [24, 25], the characteristics of the hospital [16, 26], access to health services [16] including health insurance [4, 15, 22, 27–29] and medical assistance and poverty alleviation policies [16, 23].

These CHE determinants vary across time and geographical location. The time lag between the implementation of health policy and its outcomes requires panel, or longitudinal data [30]. Based on the same definition and measurement of CHE, most CHE China studies utilized cross-sectional survey data [15, 27–29] or limited 2 or 3-year longitudinal comparison studies [24]. Only two Chinese studies used panel data from the China Family Panel Studies (CFPS) to analyse the trends of CHE incidence and intensity in China [22, 31]. Zhao et al. focused on trends and socioeconomic disparities in CHE and health impoverishment in China using 4-year panel data (2010, 2012, 2014 and 2016) from the urban and rural perspective, but only applied the 2016 data, and not the panel model, to explore the determinants affecting CHE incidence [31]. Zhao et al. reported that the proportion of households experiencing CHE decreased from 19.37 % to 2010 to 15.11 % in 2016 and the logistic regression model showed that chronic diseases, economic status, household size, residence location, age and education of the household head exert an influence on CHE. A study by Sun [29] applied a random effect model to 3 years (2012, 2014 and 2016) of panel data to reveal the CHE trends from the perspective of different BMI schemes. Sun found that total CHE incidence and intensity exhibited overall rising trends from 2012 to 2016 and the random effect model showed that households covered by BMI schemes did not decrease the odds of CHE occurrence.

We address these contradictory CHE outcomes by assessing the changes across time, location, socioeconomic household characteristics and household health utilization in the trends of CHE, and their determinants, and by analyzing China’s complex health policies. First, we construct a unique CHE 5-year nationally representative panel database using CFPS to assess the trend in the incidence and intensity of CHE. Second, employing a linear probability and random effect model, we analyze the role of the characteristics of the household head, the household socio-economics and household health utilization as determinants of CHE.

## Materials and methods

### Data

Collected by Institute of Social Science and Survey (ISSS) of Peking University, China Family Panel Studies’ (CFPS) data covers China’s society, economy, population, education and health. Our anonymized health data were obtained directly from the CFPS official website (http://www.isss.pku.edu.cn/cfps/). From five waves of the 2010–2018 CFPS, we constructed a nationally representative longitudinal database on the health and socio-economic status of households and individuals aged 16 and above across 25 provinces in China. Using a multistage probability sampling approach with implicit stratification, the 2010 CFPS baseline survey yielded a representative national sample at county level, village level, and household level [32]. In order to analyze the trends, incidence, and determinants of CHE, we built an unbalanced panel database by matching the household ID in the current CFPS survey with the same household in the previous CFPS survey. After managing missing and abnormal values, we obtained a sample consisting of 11,700 households in 2010, 9290 in 2012, 11,309 in 2014, 13,092 in 2016 and 11,520 in 2018. We constructed the panel structure by tracking 7386 or 63.12 % of the 2010 households in the 2012 sample, which provided data on the trends in the same household’s circumstances during the preceding two-year period. In the third wave survey in 2014, 64.67 % (7313/11,309) households from 2012 were tracked; in the fourth wave survey in 2016, 72.62 % (9507/13,092) households were tracked from 2014 survey and in the fifth wave survey in 2018, 82.14 % (9462/11,520) of the 2016 households were tracked.

### Variables

#### Measurement of CHE, CHE incidence and intensity

Our data are based on households, where a household member was defined in terms of marriage, blood or an adoptive relationship and an on-going economic tie. The head of the household was confirmed through the question “When your family encounters important matters and decisions have to be made, who has the final say?”. The minimum age of the household head was set as over 16 years old. Following previous research, we chose the question “In the past 12 months, the total expenditure of your family including food, clothing, housing and transportation and so on” in CFPS to measure total household expenditure; the question “Excluding the reimbursable and expected reimbursable expenses, but including the part paid by relatives and friends, your family’s medical and health expenditures last year” to measure household out-of-pocket health expenditure; and the question on monthly meal expenses to estimate yearly food expenditure, with non-food expenditure equal to total household expenditure minus food expenditure.

When a household’s out-of-pocket health payments, including outpatient and inpatient services, preventative care, maternal and child health services and medication expenses, exceed 40 % of its capacity to pay for non-food expenditure, the household faces CHE. CHE was coded 1 when OOP health expenditures as a proportion of household income, or capacity to pay (CTP), was equal or exceeded 0.4 and 0 otherwise. CHE incidence was measured by headcount, which refers to the percentage of households incurring CHE to the total household sample:

1 $$\begin{array}{*{20}c}Headcount=\frac{1}{N}\sum _{i=1}^{N}{CHE}_{i}\end{array}$$

where $N$ represents the sample size, ${CHE}_{i}$ is 1 when the ${i}_{th}$household incurred CHE, and 0 otherwise.

CHE intensity was measured by overshoot which is the degree by which an average OOP health expenditure exceeds the threshold of the total sample [16]:

2 $$\begin{array}{*{20}c}Overshoot=\frac{1}{N}\sum _{i=1}^{N}{CHE}_{i}\left(\frac{{OOP}_{i}}{{CTP}_{i}}-z\right)\end{array}$$

where $N$is the sample size, ${OOP}_{i}$ and ${CTP}_{i}$ refer to OOP health expenditure and capacity to pay of the ${i}_{th}$ household and $z$ is the threshold value, which is 40 %.

#### Independent variables

Based on the previous studies, three categories of factors influencing the household’s risk of CHE were collected. First, demographic characteristics of the household head, including sex, age (16–34 years, 35–54 years, 55–64 years and 65 + years), marital status (unmarried, married and divorced or widow), education (illiteracy and elementary, middle school, high school and college and above) and self-rated health (poor, medium and good). Second, household characteristics comprising whether there were household members aged 65 years and older; household members had a doctor-diagnosed chronic disease(s) in the past 6 months; family size (1–3, 4–5 and ≥ 6 members); and household economic status, measured by the quartile of annual per capita household income. The last category of factors refers to the healthcare utilization of the household, consisting of whether any family member used inpatient service in the past year; outpatient usage measured by whether any household member had used outpatient services in the past two weeks; and the type of health insurance, comprising NRCMS, UEBMI, URBMI, supplementary medical insurance (SMI) and no health insurance. SMI referred to all kinds of relatively high profit packages of the insurance programme, for instance, free medical service scheme for special sectors, commercial health insurance and enterprise supplementary medical insurance.

### Linear probability model

We are interested not only in the trend in the urban-rural CHE incidence, but also the reasons and the size of their effect on the trend pattern. First, we regressed the CHE incidence by urban-rural location in each year and compared the coefficients to estimate the net effect of each determinant. There are several biases when applying the coefficient from binary logit models to compare the net effect of independent variables, which could cause model misspecification and estimation errors [33]. Linear probability modelling (LPM) is a robust method to compare the coefficients in a binary outcome regression for the same model, but different samples [34–37]. To estimate the net effects of the determinants to CHE incidence by urban-rural location from 2010 to 2018, we applied LPM to conduct regressions for the sub-sample by region in each year and compare the absolute value of coefficient in the models.

### Random effect model

Given the dependent variable, CHE incidence, is a dummy variable, the binary logit model was used to estimate the determinants of CHE. Improving on the use of cross-sectional data, we built a panel dataset to better explore the determinants for the occurrence of CHE. Since there were several unchanged variables for all or most respondents, such as sex, marriage, education, a random effect panel logit model was employed to analyze the determinants of CHE based on the CFPS 2010–2018 panel data by urban-rural location:

3 $$\begin{array}{*{20}c}Log\left({P}_{it}\right)={ln}\left(\frac{{P}_{it}}{1-{P}_{it}}\right)\\ ={\beta }_{0}+{\beta }_{1}*{Householdhead}_{t}+{\beta }_{2}*{Household}_{t}+{\beta }_{3}*{Healthservice}_{t}+{\epsilon }_{it}\end{array}$$

where ${P}_{it}$ is the likelihood of CHE occurrence for ${i}_{th}$ household in the urban-rural location in the year t (t = 2010, 2012, 2014, 2016, 2018), and ${P}_{it}/1-{P}_{it}$ is the odds ratio (OR) of CHE occurrence.

## Results

### Description of rural, urban and total households

Supplementary Table 1 displays the basic characteristics of urban, rural and total sample households by year. In 2010 and 2012 males accounted for more than 70 % of the household heads, falling to broadly male-female equity from 2014 to 2018. For rural households, although the percentage of male household heads was larger than female, the disparity became smaller. For urban households, female heads exceeded males, except for 2010, for the whole sample period. Averaging roughly 50 %, the 35–54 years was always the largest age group. More than 80 % of household heads were married. Household head’s education level were relatively low. From 2012 to 2018, more than 60 % household heads perceived their health as good, which was broadly the same for urban and rural households.

Looking at household characteristics, around 25 % of households in urban area had family member(s) aged ≥ 65 years old while it was about 30 % in rural area. Similarly, around 30 % of households contained members with doctor-diagnosed chronic disease(s), roughly equal in urban and rural areas. About 50 % of households had 1–3 members and 15 % of households had 6 or more family members, but 60 % of urban households had 1–3 family members, while 20 % of rural households had 6 or more members. The economic status of households was improving, with rising per capita income levels, but urban households were economically better off than rural households.

Finally, for health service utilization, inpatient service usage gradually increased from 16.7 % to 2010 to 25.3 % in 2018, rising from 15.9 % in 2010 to 23.6 % in 2018 for urban households and from 17.4 % in 2010 to 27.6 % in 2018 for rural households. Outpatient service also rose from 34.3 % in 2010 to 60 % in 2016, before falling to 41.9 % in 2018. In urban households, outpatient services rose from 29.8 % in 2010 to 36.8 % in 2018 and from 38.3 % in 2010 to 47.2 % in 2018 in rural households. Covering rural residents, between 55 and 65 % of households were covered by NRCMS, which is consistent with estimates of NRCMS in the 2014 National Bureau of Statistics of China, the 2015 Chinese General Social Survey, 2016 China Labor-force Dynamics Survey and 2015 China Health and Nutrition Survey of between 51.28 and 68.44 %. Urban households were covered by UEBMI, which gradually rose over the 2010–2018 period; a minority of the population, around 6-9 %, were insured by URBMI from 2010 to 2018; and the coverage rate of SMI and no health insurance fell.

### CHE incidence and intensity 2010–2018

**Fig. 1:**
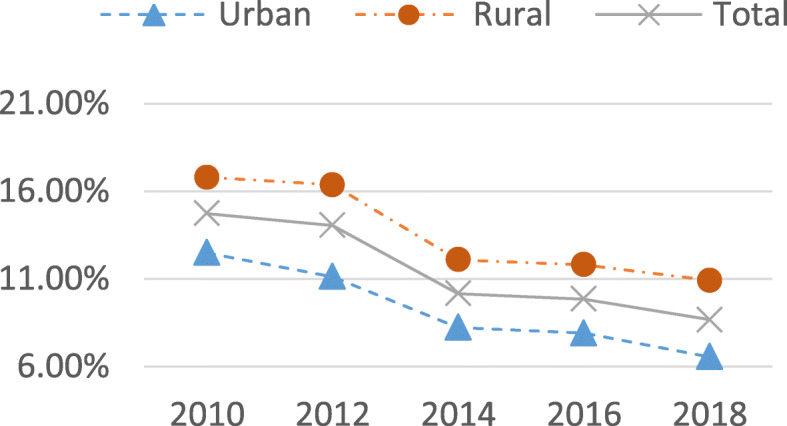
Trends of CHE headcount (Incidence) 2010-2018

Figure 1 displays the trends of CHE incidence measured by the headcount of households from 2010 to 2018. Total, urban and rural CHE incidence all showed a decreasing tendency, falling from 14.7 % in 2010 to 8.7 % in 2018 for total households, 12.5 % in 2010 to 6.6 % in 2018 for urban households and 16.8 % in 2010 to 10.9 % in 2018 for rural households. The decreasing amplitude for the 2012 to 2014 years was relatively larger than for other years.

**Fig. 2:**
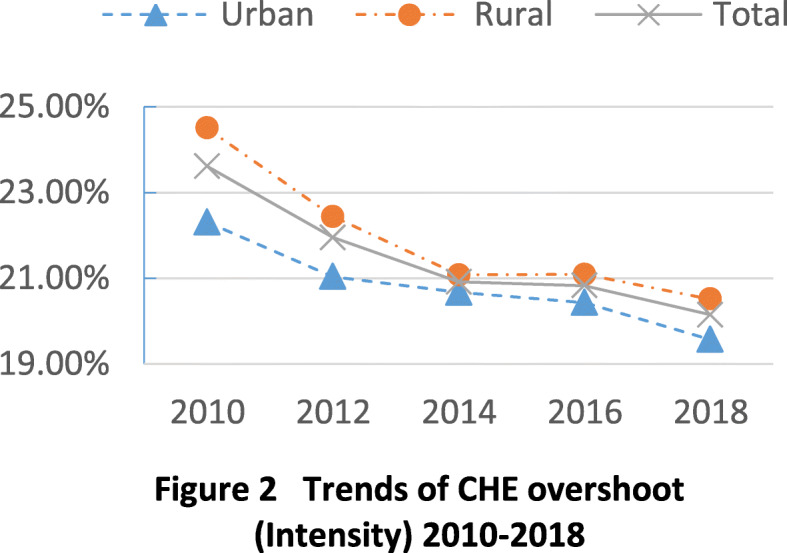
Trends of CHE overshoot (Intensity) 2010-2018

Figure 2 shows the trends of CHE overshoot, or intensity. Overall, CHE intensity decreased in rural (24.5% to 20.5%) and urban (22.3% to 19.6%) areas and for all households (23.6% to 20.2%), with the most rapid decline from 2010 to 2014.

### Determinants of CHE incidence and intensity

Supplementary Tables 2 and 3 analyse the determinants of CHE incidence in urban and rural households in each year. The linear probability model in Supplement Table 2 shows urban household head’s characteristics (age, education level, self-rated health), household characteristics (member(s) 65 + years old, family size, doctor-diagnosed chronic diseases), economic status and healthcare utilization (use of inpatient services in the past year and outpatient service use in the last two weeks and health insurance) were significant determinants of urban household CHE incidence. For rural households in Supplementary Table 3, the same variables were significant, except education and health insurance.

**Fig. 3:**
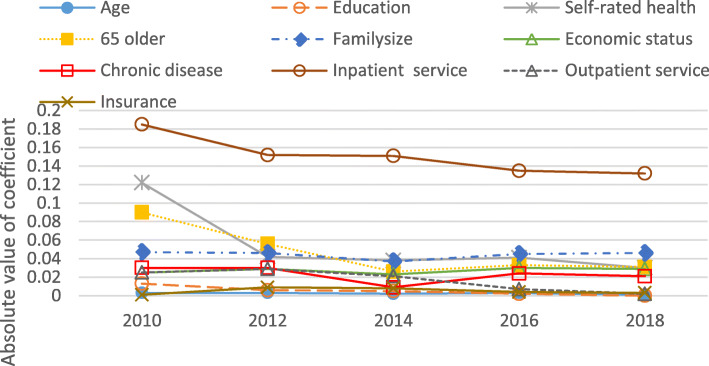
Relative Importance of determinants of CHE incidence in urban households, 2010-2018

**Fig. 4:**
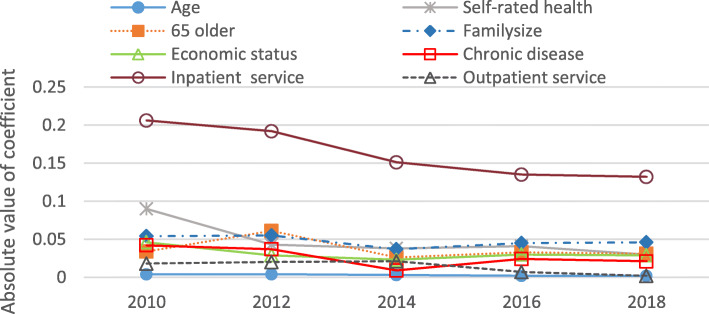
Relative Importance of determinants of CHE incidence in rural households, 2010-2018

For variables significant for at least two of the year periods, Figs. 3 and 4 show the trends in the relative importance of the determinants of urban and rural CHE incidence. For both rural and urban households, inpatient services were the most important determinant of the incidence of CHE, while household head’s age was the weakest determinant. Most determinants displayed a downward trend from 2010 to 2018, including inpatient services, self-rated health, 65 + years old household members, outpatient services, education and insurance. The relative importance of family size, economic status and chronic disease was stable in Figs. 3 and 4.

### Random effect model of CHE incidence

Table 1 presents the estimated odds ratio (OR), *p* value and corresponding 95 % confidence interval (CI) in the logit random effect model for the determinants of urban and rural CHE incidence. For urban locations, household head characteristics (age, education, self-rated health), household characteristics (members 65 years or older, chronic diseases, family size), economic status and healthcare utilization (inpatient and outpatient usage) were significant determinants of urban households’ CHE incidence. From Table 1, the same variables were significant variables for rural households, with the addition of household head’s sex and health insurance.

**Table 1:** Random effect regression of CHE incidence in urban and rural households, 2010–2018

Variables	Urban = 27,496			Rural = 28,880		
	**OR**	**95 %CI**	**P**	**OR**	**95 %CI**	**P**
Sex (Reference Female)						
Male	0.996	0.905–1.097	0.941	1.092	1.003–1.188	**0.042**
Age (Reference 16–34)						
35–54	0.944	0.780–1.143	0.556	0.919	0.791–1.068	0.272
55–64	1.706	1.398–2.083	**0.000**	1.613	1.377–1.890	**0.000**
≥ 65	2.615	2.034–3.360	**0.000**	2.087	1.712–2.535	**0.000**
Marital status (Reference Unmarried)						
Married	1.037	0.764–1.407	0.818	0.963	0.786–1.182	0.721
Divorced or widow	1.235	0.890–1.713	0.206	0.999	0.793–1.257	0.991
Education (Reference Illiterate and elementary)						
Middle school	0.904	0.806–1.014	0.086	0.852	0.785–0.924	**0.000**
High school and above	0.735	0.632–0.855	**0.000**	0.789	0.678–0.918	**0.002**
Self-rated health (Reference good)						
Poor	2.437	2.154–2.758	**0.000**	1.933	1.754–2.130	**0.000**
Medium	1.135	1.187–1.525	**0.000**	1.292	1.166–1.431	**0.000**
65 and older (Reference No)						
Yes	1.389	1.181–1.634	**0.003**	1.214	1.076–1.369	**0.002**
Chronic disease (Reference No)						
Yes	1.314	1.190–1.451	**0.000**	1.301	1.200–1.410	**0.000**
Family size (Reference 1–3)						
4–5	0.608	0.543–0.680	**0.000**	0.608	0.556–0.665	**0.000**
≥ 6	0.509	0.431-0.600	**0.000**	0.468	0.420–0.521	**0.000**
Economic status (Reference Quartile 4 (Highest))						
Quintile 1 (Lowest)	2.655	2.296–3.070	**0.000**	2.253	1.948–2.605	**0.000**
Quintile 2	1.763	1.528–2.035	**0.000**	1.572	1.354–1.825	**0.000**
Quintile 3	1.519	1.334–1.729	**0.000**	1.218	1.043–1.421	**0.013**
Inpatient service (Reference No)						
Yes	3.710	3.367–4.087	**0.000**	3.395	3.134–3.677	**0.000**
Outpatient service (Reference No)						
Yes	1.236	1.121–1.363	**0.000**	1.145	1.057–1.240	**0.001**
Health insurance (Reference No insurance)						
NRCMS	1.084	0.926–1.270	0.315	1.164	1.005–1.349	**0.043**
UEBMI	0.932	0.782–1.111	0.434	0.865	0.654–1.144	0.308
URBMI	1.181	0.985–1.416	0.073	1.139	0.833–1.558	0.414
SMI	0.929	0.756–1.142	0.485	0.898	0.677–1.190	0.453
cons	0.011	0.007–0.018	0.000	0.043	0.028–0.065	0.000
lnsig2u	-2.028	-3.298–0.758	——	-2.128	-3.390–0.866	——
sigma_u	0.363	0.192–0.685	——	0.345	0.184–0.649	——
rho	0.038	0.011–0.125	——	0.035	0.010–0.113	——
Prob > chi2	0.000			0.000		
Chi-square	186.94			263.36		
AIC	13611.27			19462.716		

For urban households, household heads aged 55–64 group were 1.706 times and the 65 + age group were 2.615 times more likely to incur CHE than the 16–34 year old reference group. High school and above education level of the household head decreased the risk of CHE from 1 to 0.735; the poor were 2.437 times more likely to incur CHE; and the medium health group 1.135 times more likely to incur CHE. Households with family members 65 + years old were 1.389 times, and those with doctor-diagnosed chronic disease 1.314 times, more likely to suffer CHE. Larger size households with 4–5 members decreased the odds of suffering CHE from 1 to 0.608 and 6 + members households to 0.509. Households in lowest quartile income group was 2.655 times more likely, quartile 2 income group was 1.763 times more likely, and quartile 3 group was 1.519 times was more likely to incur CHE. Inpatients were 3.710 times more likely, and outpatients were 1.236 times more likely, to suffer CHE. Finally, the health insurance variable was not significant.

For rural households, household heads aged 55–64 were 2.087 times more likely and aged 65 + 1.613 times more likely to incur CHE than the 16–24 reference group. Mirroring the results for urban areas, high levels of household head education effectively decreased the risk of CHE. The self-rated poor health group was 1.933 times more likely and the medium health group 1.292 times more likely to experience CHE. Households with family members 65+ years  were 1.214 times more likely, and those with doctor-diagnosed chronic diseases was 1.301 times more likely, to incur CHE. Similar to urban households, more family members reduced the risk of CHE, while poor income status increased the probability of CHE. Inpatients were 3.395 times more likely, and outpatients were 1.145 times more likely, to suffer CHE. Sex was an especially important determinant of rural CHE, where male household heads were 1.092 times more likely to suffer CHE.Compared with households covered by no insurance, those covered by NRCMS was 1.164 times more likely to incur CHE. Other insurance groups showed no effect on CHE occurrence.

## Discussion

Our study applied a 5-year nationally representative Chinese household panel dataset and a conservative method to analyze the trends, incidence, intensity and determinants of CHE for urban and rural areas in China. Measured by headcount, CHE incidence decreased from 14.73 % in 2010 to 8.67 % in 2018, with CHE in rural areas about 4 % higher than for urban households. CHE intensity, measured by overshoot, also fell from 23.61 % in 2010 to 20.15 % in 2018, with rural areas about 1 % higher than urban households. Using the same measurement of CHE as previous studies, our results were broadly consistent with these studies [38, 39], which found the overall incidence of CHE was around 13 %, displayed a downward trend and was higher in rural than urban areas. But our estimates were lower than Sun’s CHE estimates, who also used the CFPS panel data [22], this might be due to Sun’s balanced panel data dropping almost half the observations in his samples each year. Our higher CHE in rural areas than urban areas was consistent with other studies [25, 31, 40], with some international studies reporting that rural households’ incidence of CHE over 1.5 times higher than the national average [31, 41].

There were a number of possible factors explaining these outcomes. First, the decreasing 2010–2018 CHE trend was a result of a combination effects from economic growth and various policy interventions. Due to economic growth, CTP grew at 360.2 % and household income per capita grew at 168.4 %, compared to the 64.63 % growth rate of OOP expenses, which reduced the ratio of OOP to CTP and the incidence of CHE. Second, policy interventions reduced CHE, especially the expansion of universal health insurance coverage and enhanced allowances for vulnerable populations, such as elderly people[42]. Third, our 13 % incidence estimate of CHE was relatively high compared to other countries, which may reflect China’s fee-for-service payment system and low level of benefit packages [31]. Fourth, given the disparities in economic level, the social security system and health resources between urban and rural areas in China, CHE remained higher in rural than urban households [43].

Our linear probability model and random effect model provided new insights into CHE in China. The relative importance of household head’s characteristics (age, education level, self-rated health), household characteristics (member(s) 65 + years old, family size, economic status, doctor-diagnosed chronic diseases) and healthcare utilization (use of inpatient services in the past year and outpatient service use in the last two weeks and health insurance) as determinants of CHE incidence declined over the 2010–2018 period, with the decline about the same in urban and rural areas. Inpatient service usage accounted for 10–20 % of all determinants of CHE, and addressing the costs of inpatient service usage would attenuate CHE in China.

The random effect model revealed that age, education, self-rated health of household head, members 65 years or older, chronic diseases, family size, economic status and urban healthcare utilization were significant determinants of urban households’ CHE incidence. The same variables were significant for rural households, with the addition of household head’s sex and health insurance. Previous studies also showed age and chronic diseases of family member increased the probability of CHE [24–26, 31, 44, 45]. These trends highlight the urgency in developing long-term care insurance, extending insurance coverage, improving the medical health assistance system, prioritizing outpatient services, supporting the costs of essential medications and expanding to rehabilitation services [31].

We found that large household size protectsed against CHE, which was also reported in some other studies [16, 46]. Larger households could share the CHE economic risk among a larger number of people that meant greater family-based medical cost affordability [16]. We also found that households in poor income quartiles more likely to suffer CHE, which is consistent with other Chinese and international studies [31, 47]. These results emphasize that reform to China’s social security system and income equality policies could provide a safety net for poor households, the rural elderly, those with chronic diseases and single member households. The government should consider establishing subsidiary health insurance funds and a financial rescue system that attenuates the economic burden of disease for low-income households before they are pushed into the “poverty trap”.

A key finding was that health service utilization was the single most important variable both in urban and rural areas, while most previous studies only included inpatient services [46] and the two studies using CFPS panel data did not include any health service utilization variable [22, 26, 31]. In China, consultation visits to medical institutions grew sharply from 5.8 billion visits in 2010 to 8.3 billion visits in 2018, and the annual hospitalization rate jumped from 10.5 % in 2010 to 18.2 % in 2018 [48]. Much of this growth in health demand, and greater CHE risk, can be traced to China’s distorted health care incentive mechanisms that encouraged over-servicing. Based on pilot hospital reforms experience, such as diagnosis-related groups (DRGs), canceling drug bonuses, lowering the prices of medical consumables, examinations and large medical equipment testing, we propose that the government should continue an urgent assessment of the costs-benefits of these reforms.

We identified significant differences in CHE risk between rural and urban households, including household head sex and health insurance. Previous studies took mainly a nationwide view of health insurance and CHE, with contradictory results [2, 4, 16, 27, 29, 39]. Most importantly, more than 80 % of the rural households were covered by NRCMS, which provided more limited protection against CHE compared to UEBMI [43]. Government should address the fragmented BMI schemes, aligning the benefit schedules.

We recommended a coordinated approach to CHE reform. The government should continue to promote economic growth and to accelerate the financing level and risk pooling of the BMI schemes, especially for NRCMS, to enhance the reimbursement ratio and provide fuller outpatient service coverage. To improve the payment capacity of medical insurance funds, we suggest the current funds, especially NRCMS, should be financed at the city-province level not the county-level. Second, the integration of BMI schemes should be promoted at the national level to eliminate the fragmentation. Third, the CMI list should be broadened and based on treatment needs in relation to catastrophic illness and pharmaco-economics evaluations instead of following the BMI. Finally, further assessment of hospital payment reform, reimbursement policy reform and hospital innovations, such as DRGs, should be expedited to determine the efficacy of their roll-out across the hospital system.

## Limitations

There are several limitations. The standard CHE calculation used here excluded extremely poor households that cannot afford health services. Future research should identify families so poor they did not access a doctor. Second, OOP health expenditure in CFPS survey did not cover the non-medical direct and indirect cost for medical services, such as transportation expense, accommodation costs and income loss, which may lead to an underestimate of the incidence and intensity of CHE. Third, the absence of data on the characteristics of hospitals, such as hospital level, should be addressed in future studies. Fourth, the situation where adult children support their parents by paying for their medical care, or vice versa, should be addressed in future studies. Finally, CFPS health service utilization, health expenditure and household income were self-reported, which may be less accurate than data from medical records.

## Conclusions

CHE incidence decreased from 2010 to 2018 both in urban and rural areas, but CHE was higher in rural than urban households. For both urban and rural households, inpatient service usage remained the most important determinant of CHE incidence, while age was the weakest factor. Almost all the included variables influenced CHE incidence in urban households (except gender and marriage) and rural household (except marriage). Both in urban and rural areas, the older household head’s age, poor and medium self-rated health, family members 65 + years, members with doctor-diagnosed chronic diseases, low household income status, inpatient and outpatient service utilization significantly increased the risk of suffering CHE. There were differences in association with CHE risk between rural and urban households, including household head sex, education level and type of health insurance. These findings have important policy implications for healthcare delivery and financing in China and other developing countries. To address CHE, the policy implications include improving the economic level of poor households and spreading equitably the per capita income benefits of economic growth; further reforming national health insurance schemes, especially improving the benefit package of public basic insurance, and improving commercial insurance and specific insurance schemes for vulnerable populations; and reinforcing pre-payment hospital insurance methods to control OOP hospital expenses.

## Supplementary Information

**Additional file 1:**

## Data Availability

The datasets used in this study were derived from China Family Panel Studies in the years of 2010,2012,2014,2016 and 2018. Available at: http://www.isss.pku.edu.cn/cfps/. Accessed 19 Aug 2019. The code used in this study is available from the corresponding author on reasonable request.

## Abbreviations

CHE: Catastrophic health expenditures
OOP: Out-of-pocket payments
CFPS: China Family Panel Studies
CTP: Capacity to pay
BMI: Basic medical insurance
UEBMI: Urban employee basic medical insurance
URBMI: Urban resident basic medical insurance
NRCMS: New rural cooperative medical scheme
CMI: Catastrophic medical insurance
SMI: Supplement medical insurance
DRGs: Diagnosis related groups
LPM: Linear probability modelling
OR: Odds ratio
CI: Confidence interval

## References

[1] Zhu W, Xia Y. An analysis on household consumption-fueled borrowing in China. J Finance Econ. 2018;44(10):67–81. 10.16538/j.cnki.jfe.2018.10.005.

[2] Xu K, Evans DB, Kawabata K, et al. Household catastrophic health expenditure: a multicountry analysis. Lancet. 2003;362:111–7. 10.1016/S0140-6736(03)13861-5. 10.1016/S0140-6736(03)13861-5 12867110

[3] Wagstaff A, Van Doorslaer E. Catastrophic and impoverishment in paying for health care: with application to Vietnam 1993-98. Health Econ. 2003;12:921–34. 10.1002/hec.776. 10.1002/hec.776 14601155

[4] Wagstaff A, Lindelow M. Can insurance increase financial risk? The curious case of health insurance in China. J Health Econ. 2008;27(4):990–1005. 10.1016/j.jhealeco.2008.02.002. 10.1016/j.jhealeco.2008.02.002 18342963

[5] Dorjdagva J, Batbaatar E, Svensson M, Dorjsuren B, Kauhanen J. Catastrophic health expenditure and impoverishment in Mongolia. Int J Equity Health. 2016;15:105. 10.1186/s12939-016-0395-8. 10.1186/s12939-016-0395-8 PMC4939814 27401464

[6] Chuma J, Maina T. Catastrophic health care spending and impoverishment in Kenya. BMC Health Serv Res. 2012;12:413. 10.1186/1472-6963-12-413. 10.1186/1472-6963-12-413 PMC3561146 23170770

[7] Amaya-Lara JL (2016). Catastrophic expenditure due to out-of-pocket health payments and its determinants in Colombian households. Int J Equity Health.

[8] World Health Organization. Global Health Expenditure Database. https://apps.who.int/nha/database/Home/Index/zh. Accessed 10 Mar 2021.

[9] National Health Commission of the People’s Republic of China. Study report on total health expenditure in China. http://www.nhc.gov.cn/guihuaxxs/s10748/202006/ebfe31f24cc145b198dd730603ec4442.shtml. Accessed 02 May 2021.

[10] Li Y, Wu Q, Xu L, Legge D, Hao Y, Gao L (2012). Factors affecting catastrophic health expenditure and impoverishment from medical expenses in China: policy implications of universal health insurance. Bull World Health Organ.

[11] Yin W. To accelerate the development of people’s health and promote the innovation of medical insurance system. In: People’s Daily. 2016. http://healthpeople.com.cn/n1/2016/1010/c398004-28764621.html. Accessed 13 June 2020.

[12] He A, Wu S. Towards universal health coverage via social health insurance in China: systemic fragmengtation, reform imperatives, and policy alternatives. Appl Health Econ Health Policy. 2017;15:707–16. 10.1007/s40258-016-0254-1. 10.1007/s40258-016-0254-1 27333794

[13] Meng Q, Fang H, Liu X, Yuan B, Xu J (2015). Consolidating the social health insurance schemes in China: towards an equitable and efficient health system. Lancet.

[14] Pan X, Xu J, Meng Q. Integrating social health insurance systems in China. Lancet. 2016;387:1274–75. 10.1016/S0140-6736(16)30021-6. 10.1016/S0140-6736(16)30021-6 27025430

[15] Zhao S, Zhang X, Dai W, Ding Y, Chen J, Fang, P. Effect of the catastrophic medical insurance on household catastrophic health expenditure: evidence from China. Gaceta Sanitaria. 2020;34(4):370–76. 10.1016/j.gaceta.2018.10.005. 10.1016/j.gaceta.2018.10.005 30704817

[16] Wang Z, Li X, Chen S. Catastrophic health expenditures and its inequality in elderly households with chronic disease patients in China. Int J Equity Health. 2015;14:8. 10.1186/s12939-015-0134-6. 10.1186/s12939-015-0134-6 PMC4304672 25599715

[17] Kien VD, Minh HV, Giang KB, et al. Socioeconomic inequalities in catastrophic health expenditure and impoverishment associated with non-communicable diseases in urban Hanoi, Vietnam. Int J Equity Health. 2016;15:169. 10.1186/s12939-016-0460-3. 10.1186/s12939-016-0460-3 PMC5064924 27737663

[18] Alkhatib AA, Hilden K, Adler DG. Impact of the policy of expanding benefit coverage for cancer patients on catastrophic health expenditure across different income groups in South Korea. Soc Sci Med. 2015;138:241–7. 10.1016/j.socscimed.2015.06.012. 10.1016/j.socscimed.2015.06.012 26123883

[19] Rahman M, Gilmour S, Saito E, Sultana P, Shibuya K (2013). Health-related financial catastrophe, inequality and chronic illness in Bangladesh. PLoS One.

[20] Leng A, Li J, Nicholas S, Wang J. Catastrophic health expenditure of cancer patients at the end-of-life: a retrospective observational study in China. BMC Palliative Care. 2019;18(1):43. 10.1186/s12904-019-0426-5. 10.1186/s12904-019-0426-5 PMC6533646 31122235

[21] Guan X, Li H, Xin X, Guo Z, Ma L, Han S, et al. Research on equity and influential factors of medicine expenditure affordability for diabetes patients in China. Chin Pharm Aff. 2015;29:1047–54. in Chinese. 10.16153/j.1002-7777.2015.10.010.

[22] Sun J, Lyu SJ. The effect of medical insurance on catastrophic health expenditure: evidence from China. Cost Eff Resour Alloc. 2020;18(10):1–11. 10.1186/s12962-020-00206-y. 10.1186/s12962-020-00206-y PMC7045636 32127784

[23] Zhang X, Xu Q, Xu L, Jing Z, Sun L, Li J, Zhou C. Catastrophic health expenditure: a comparative study between hypertensive patients with and without complication in rural Shandong, China. BMC Public Health. 2020;20(1):545. 10.1186/s12889-020-08662-0. 10.1186/s12889-020-08662-0 PMC7178564 32321485

[24] Quintal C. Evolution of catastrophic health expenditure in a high income country: incidence versus inequalities. Int J Equity Health. 2019(18):145–56. 10.1186/s12939-019-1044-9. 10.1186/s12939-019-1044-9 PMC6749702 31533723

[25] Yazdi-Feyzabadi V, Bahrampour M, Rashidian A, Haghdoost AA, Javar MA, Mohammad Hossein Mehrolhassani. Prevalence and intensity of catastrophic health care expenditures in Iran from 2008 to 2015: a study on Iranian household income and expenditure survey. Int J Equity Health. 2018;17(1):44. 10.1186/s12939-018-0743-y. 10.1186/s12939-018-0743-y PMC5899413 29653568

[26] Liu H, Zhu H, Wang J, Qi X, Zhao M, Shan L, et, al. Catastrophic health expenditure incidence and its equity in China: a study on the Initial implementation of the medical insurance integration system. BMC Public Health. 2019;19(1):1761. 10.1186/s12889-019-8121-2. 10.1186/s12889-019-8121-2 PMC6937839 31888591

[27] Jue Y, Yong L, Nina H. Empirical study on the relief effect of catastrophic health expenditure under three basic medical schemes. Chin Health Econ. 2012;31(1):26–8. in Chinese. 10.3969/j.issn.1003-0743.2012.01.008.

[28] Wang Y, Xu D. Can basic medical insurance reduce catastrophic health spending for residents? Evidence from data of CHARLS. Financ Theory Pract. 2019;41(2):87–94. in Chinese 10.3969/j.issn.1003-4625.2019.02.012.

[29] Guo N, Iversen T, Lu M, Wang J, Shi L. Does the new cooperative medical scheme reduce inequality in catastrophic health expenditure in rural China? BMC Health Serv Res. 2016;16(1):653. 10.1186/s12913-016-1883-7. 10.1186/s12913-016-1883-7 PMC5214924 28052775

[30] Cheng H, Augustin D, Glass EH, Anthony JC. Nation-scale primary prevention to reduce newly incident adolescent drug use: the issue of lag time. Peer. 2019;7(11):e6356. 10.7717/peerj.6356. 10.7717/peerj.6356 PMC6376940 30775172

[31] Zhao Y, Oldenburg B, Mahal A, Lin Y,Tang S, Liu X. Trends and socio-economic disparities in catastrophic health expenditure and health impoverishment in China: 2010 to 2016. Trop Med Int Health. 2019;25(2):236–47. 10.1111/tmi.13344. 10.1111/tmi.13344 31713972

[32] Xie Y, Lu P. The sampling design of the China Family Panel Studies (CFPS). Chin J Sociol. 2015:1(4):471–84. 10.1177/2057150X15614535. 10.1177/2057150X15614535 PMC5973535 29854418

[33] Hoetker, Glenn P. Confounded coefficients: Extending recent advances in the accurate comparison of logit and probit coefficients across groups. Working paper. Oct. 22, 2004. University of Illinoisat urbana-chanpaign. www.Public.Asu.edu/…/research/Hoetker-confounded.wp.pdf. 10.2139/ssrn.609104.

[34] Wooldridge Jeffery M (2002). Econometric analysis of cross sectional and panel data.

[35] Mood C. Logistic regression: why we cannot do what we think we can do, and what can do about it. Eur Sociol Rev. 2010;26(1):67–82. 10.2307/40602478.

[36] Karlson, Kristian B, Anders H, Richard B. Comparing regression coefficients between same-samples nested models using logit and probit: a new method. Sociol Methodol. 2013;42(1): 286–313. 10.1177/0081175012444861.

[37] Hong Y. On the coefficients comparison between logistic regression and the solutions: a brief review. Chin J Sociol. 2015;35(04):227–48. In Chinese. 10.15992/j.cnki.31-1123/c.2015.04.009.

[38] Wu D, Yu F (2018). Improvement of the reduction in catastrophic health expenditure in China’s public health insurance. PLoS ONE.

[39] Meng Q, Xu L, Zhang Y, Qian J, Cai M, Xin Y, Gao J, Xu K, Boerma JT, Barber SL. Trends in access to health services and financial protection in China between 2003 and 2011: a cross-sectional study. Lancet. 2012: 379(9818): 805–14. 10.1016/S0140-6736(12)60278-5. 10.1016/S0140-6736(12)60278-5 22386034

[40] Abu-Zaineh M, Romdhane HB, Ventelou B, Moatti JP, Chokri A. Appraising financial protection in health: the case of Tunisia. Int J Health Care Finance Econ. 2013;13(1):73–93. 10.1007/s10754-013-9123-8. 10.1007/s10754-013-9123-8 23381233

[41] O’Donnell O, Doorslaer E, Rannan-Eliya A, Somanathan C, Doorslaer E, Rannan-Eliya S. Explaining the incidence of catastrophic payments for health care: comparative evidence from Asia. EQUITAP Working Paper No. 5. 2005.

[42] Embassy of the People’s Republic of China in the Federal Republic of Germany. China’s health care reform. http://www.fmprc.gov.cn/ce/cede/det/dshd/t787403.htm. Accessed 17 Jan 2011.

[43] Chen Z, Jiang Y, Li W, Chen Z, Jiang Y, Li W (2016). Does NRCMS reduce the incidence and intensity of catastrophic out-of -pocket health expenditure? Based on universal coverage of CRCMS. Finan Econ.

[44] Arsenijevic J, Pavlova M, Rechel B, Groot W (2016). Catastrophic health care expenditure among older people with chronic diseases in 15 European countries. PLoS One.

[45] Wang J, Chen L, Ye T, Zhang Z, Ma J. Financial protection effects of modification of China’s New Cooperative Medical Scheme on rural households with chronic diseases. BMC Health Serv Res. 2014;14(1):305. 10.1186/1472-6963-14-305. 10.1186/1472-6963-14-305 PMC4107472 25023600

[46] Wang J, Zhu H, Liu H, Wu K, Zhang X, Zhao M, et al. Can the reform of integrating health insurance reduce inequity in catastrophic health expenditure? Evidence from China. Int J Equity Health. 2020;19(1):49. 10.1186/s12939-020-1145-5. 10.1186/s12939-020-1145-5 PMC7126184 32245473

[47] Su T, Kouyaté B, Flessa S. Catastrophic household expenditure for health care in a low income society: a study from Nouna district, Burkina Faso. Bull World Health Organ. 2006;84:21–7. 10.1590/S0042-96862006000100010. 10.2471/blt.05.023739 PMC2626518 16501711

[48] National Health Commission of the People’s Republic of China. Bulletin of national health commission of the People’s Republic of China. http://www.nhc.gov.cn/wjw/gongb/list.shtml. Accessed 22 May 2019.

