# Supplementary material for: Trends and determinants of catastrophic health expenditure in China 2010–2018: a national panel data analysis
Source: BMC Health Serv Res. 2021 May 29;21:526. doi: 10.1186/s12913-021-06533-x (PMC8164806; doi:10.1186/s12913-021-06533-x)
Supplement: Supplementary file 1 — Additional file 1: [file 12913_2021_6533_MOESM1_ESM.docx]

**Supplementary Table 1 Characteristics of urban, rural and total households from 2010 to 2018（N (%)）**

| **VariableVriables** | **2010** | |  |  | **2012** | |  |  | **2014** | |  |  | **2016** | |  |  | **2018** | |  |
| --- | --- | --- | --- | --- | --- | --- | --- | --- | --- | --- | --- | --- | --- | --- | --- | --- | --- | --- | --- |
|  | **Urban** | **Rural** | **Total** |  | **Urban** | **Rural** | **Total** |  | **Urban** | **Rural** | **Total** |  | **Urban** | **Rural** | **Total** |  | **Urban** | **Rural** | **Total** |
| Household Head  Sex |  |  |  |  |  |  |  |  |  |  |  |  |  |  |  |  |  |  |  |
| Male | 3,711(66.1) | 4,966(81.6) | 8,677(74.2) |  | 2,811(31.3) | 4,164(80.1) | 6,975(75.1) |  | 2,765(49.0) | 3,317(58.5) | 5,227(46.2) |  | 3,114(47.7) | 3,714(56.6) | 6,828(52.2) |  | 2,922(49.4) | 3,080(54.9) | 6,002(52.1) |
| Female | 1,903(33.9) | 1,120(18.4) | 3,023(25.8) |  | 1,282(68.7) | 1,033(19.9) | 2,315(24.9) |  | 2,877(51.0) | 2,350(41.5) | 6,082(53.8) |  | 3,417(52.3) | 2,847(43.4) | 6,264(47.9) |  | 2,990(50.6) | 2,528(45.1) | 5,518(47.9) |
| Age |  |  |  |  |  |  |  |  |  |  |  |  |  |  |  |  |  |  |  |
| 16-34 | 640(11.4) | 550(9.0) | 1,190(10.2) |  | 412(10.1) | 505(9.7) | 917(9.9) |  | 1,021(18.1) | 712(12.6) | 1,733(15.3) |  | 1,434(22.0) | 1,029(15.7) | 2,463(18.8) |  | 1,366(23.1) | 844(15.1) | 2,210(19.2) |
| 35-54 | 2,879(51.3) | 3,384(55.6) | 6,263(53.5) |  | 2,058(50.3) | 2,720(52.3) | 4,778(51.4) |  | 2,616(46.4) | 2,824(49.8) | 5,440(48.1) |  | 2,787(42.7) | 3,045(46.4) | 5,832(44.6) |  | 2,490(42.1) | 2,414(43.1) | 4,904(42.6) |
| 55-64 | 1,243(22.1) | 1,369(22.5) | 2,612(22.3) |  | 927(22.7) | 1,217(23.4) | 2,144(23.1) |  | 1,103(19.6) | 1,206(21.3) | 2,309(20.4) |  | 1,166(17.9) | 1,299(19.8) | 2,465(18.8) |  | 1,015(17.2) | 1,235(22.0) | 2,250(19.5) |
| ≥65 | 852(15.2) | 783(12.9) | 1,635(14.0) |  | 696(17.0) | 755(14.5) | 1,451(15.6) |  | 902(16.0) | 925(16.3) | 1,827(16.2) |  | 1,144(17.5) | 1,188(18.1) | 2,332(17.8) |  | 1,041(17.6) | 1,115(19.9) | 2,156(18.7) |
| Marital status |  |  |  |  |  |  |  |  |  |  |  |  |  |  |  |  |  |  |  |
| Unmarried | 118(2.1) | 154(2.5) | 272(2.3) |  | 90(2.2) | 148(2.9) | 238(2.6) |  | 325(5.8) | 224(4.0) | 549(4.9) |  | 470(7.2) | 380(5.8) | 850(6.5) |  | 492(8.3) | 312(5.6) | 804(7.0) |
| Married | 4,899(87.3) | 5,481(90.1) | 10,380(88.7) |  | 3,552(86.8) | 4,652(89.5) | 8,204(88.3) |  | 4,758(84.3) | 4,996(88.2) | 9,754(86.3) |  | 5,373(82.3) | 5,571(84.9) | 10,944(83.6) |  | 4,832(81.7) | 4,728(84.3) | 9,560(83.0) |
| Divorced or widow | 597(10.6) | 451(7.4) | 1,048(9.0) |  | 451(11.0) | 397(7.6) | 848(9.1) |  | 559(9.9) | 447(7.9) | 1,006(8.9) |  | 688(10.5) | 610(9.3) | 1,298(9.9) |  | 588(10.0) | 568(10.1) | 1,156(10.0) |
| Education Level |  |  |  |  |  |  |  |  |  |  |  |  |  |  |  |  |  |  |  |
| Illiterate and elementary | 1,892(33.7) | 3,779(62.1) | 5,671(48.5) |  | 1,545(37.8) | 3,227(62.1) | 4,772(51.4) |  | 1,914(33.9) | 3,449(60.9) | 5,363(47.4) |  | 2,184(35.0) | 3,874(61.2) | 6,058(48.2) |  | 1,719(29.1) | 3,205(57.2) | 4,924(42.7) |
| Middle school | 1,823(32.5) | 1,717(28.2) | 3,540(30.3) |  | 1,266(30.9) | 1,394(26.8) | 2,660(28.6) |  | 1,742(30.9) | 1,592(29.1) | 3,334(29.5) |  | 1,832(29.4) | 1,656(26.1) | 3,488(27.7) |  | 1,798(30.4) | 1,643(29.3) | 3,441(29.9) |
| High school and above | 1,899(33.8) | 590(9.7) | 2489(21.2) |  | 1281(31.3) | 576(11.1) | 1,857(20.0) |  | 1,986(35.2) | 626(11.0) | 2612(23.1) |  | 2222(35.6) | 804(12.7) | 3026(24.1) |  | 2395(40.5) | 760(12.5) | 3155(27.4) |
| Self-rated health |  |  |  |  |  |  |  |  |  |  |  |  |  |  |  |  |  |  |  |
| Poor | 85(1.5) | 150(2.5) | 235(2.0) |  | 696(17.0) | 1,109(21.3) | 1,805(19.4) |  | 791(14.0) | 1,124(19.8) | 1,915(16.9) |  | 914(14.0) | 1,301(19.8) | 2,215(16.9) |  | 868(14.7) | 1,194(21.3) | 2,062(17.9) |
| Medium | 364(6.5) | 674(11.1) | 1,038(8.9) |  | 906(22.1) | 943(18.2) | 1,849(19.9) |  | 959(17.0) | 880(15.5) | 1,893(16.3) |  | 1,299(19.9) | 1,252(19.1) | 2,551(19.5) |  | 867(14.7) | 757(13.5) | 1,624(14.1) |
| Good | 5,165(92.0) | 5,262(86.5) | 10,427(89.1) |  | 2,491(60.9) | 3,145(60.5) | 5,636(60.7) |  | 3,892(69.0) | 3,663(64.6) | 7,555(66.8) |  | 4,318(66.1) | 4,008(61.1) | 8,326(63.6) |  | 4,177(70.7) | 3,657(65.2) | 7,834(68.0) |
| ***Household characteristics*** |  |  |  |  |  |  |  |  |  |  |  |  |  |  |  |  |  |  |  |
| 65 and order |  |  |  |  |  |  |  |  |  |  |  |  |  |  |  |  |  |  |  |
| Yes | 1,266(22.6) | 1,421(23.4) | 2,687(23.0) |  | 1,053(25.7) | 1,377(26.5) | 2,430(26.2) |  | 1,443(25.6) | 1,666(29.4) | 3,109(27.5) |  | 1,957(30.0) | 2,222(33.9) | 4,179(31.9) |  | 1,513(25.6) | 1,657(29.6) | 3,170(27.5) |
| No | 4,348(77.6) | 4,665(76.7) | 9,013(77.0) |  | 3,040(74.3) | 3,820(73.5) | 6,860(73.8) |  | 4,199(74.4) | 4,001(70.6) | 8,200(72.5) |  | 4,574(70.0) | 4,339(66.1) | 8,913(68.1) |  | 4,399(74.4) | 3,951(70.5) | 8,350(72.5) |
| Chronic disease |  |  |  |  |  |  |  |  |  |  |  |  |  |  |  |  |  |  |  |
| Yes | 1,502(26.8) | 1,739(28.6) | 3,241(27.7) |  | 1,502(26.8) | 1,342(25.8) | 2,453(26.4) |  | 1,819(32.2) | 1,791(31.6) | 3,610(31.9) |  | 2,063(31.6) | 2,134(32.5) | 4,197(32.1) |  | 1,758(29.7) | 1,823(32.5) | 3,581(31.1) |
| No | 4,112(73.3) | 4,347(71.4) | 8,459(72.3) |  | 4,112(73.3) | 3,855(74.2) | 6,837(73.6) |  | 3,823(67.8) | 3,876(68.4) | 7,699(68.1) |  | 4,468(68.4) | 4,427(67.5) | 8,895(67.9) |  | 4,154(70.3) | 3,785(67.5) | 7,939(68.9) |
| Family size |  |  |  |  |  |  |  |  |  |  |  |  |  |  |  |  |  |  |  |
| 1-3 | 3,477(61.9) | 2,252(37.0) | 5,729(49.0) |  | 2,408(58.8) | 2,087(40.2) | 4,495(48.4) |  | 3,403(60.3) | 2,423(42.7) | 5,829(51.5) |  | 3,915(59.9) | 2,952(45.0) | 6,867(52.5) |  | 3,664(62.0) | 2,747(49.0) | 6,411(55.7) |
| 4-5 | 1,718(30.6) | 2,654(43.6) | 4,372(37.3) |  | 1,287(31.5) | 2,017(38.8) | 3,304(35.6) |  | 1,699(30.1) | 2,095(37.0) | 3,794(33.6) |  | 1,906(29.2) | 2,223(33.9) | 4,129(31.5) |  | 1,621(27.4) | 1,743(31.1) | 3,364(29.2) |
| ≥6 | 419(7.5) | 1,180(19.4) | 1,599(13.7) |  | 398(9.7) | 1,093(21.0) | 1,491(16.0) |  | 540(9.6) | 1,149(20.3) | 1,689(14.9) |  | 710(10.9) | 1,386(21.1) | 2,096(16.0) |  | 627(10.61) | 1,118(19.94) | \| 1,745(15.1) \| \| --- \| |
| Economic status (quartile) |  |  |  |  |  |  |  |  |  |  |  |  |  |  |  |  |  |  |  |
| 1 (Lowest) | 1,414(25.2) | 3,178(52.2) | 4,592(39.3) |  | 935(22.8) | 2,355(45.3) | 3,290(35.4) |  | 898(15.9) | 1,847(32.6) | 2,745(24.3) |  | 572(8.8) | 1,661(25.3) | 2,233(17.1) |  | 384(6.5) | 1,278(22.8) | 1,662(14.4) |
| 2 | 1,573(28.0) | 1,828(30.0) | 3,401(29.1) |  | 925(22.6) | 1,318(25.4) | 2,243(24.1) |  | 986(17.5) | 1,485(26.2) | 2,471(21.8) |  | 972(14.9) | 1,784(27.2) | 2,756(21.1) |  | 678(11.5) | 1,504(26.8) | 2,182(18.9) |
| 3 | 1,398(24.9) | 809(13.3) | 2,207(18.9) |  | 1,146(28.0) | 1,071(20.6) | 2,217(23.9) |  | 1,522(27.0) | 1,474(26.0) | 2,996(26.5) |  | 1,559(23.9) | 1,758(26.8) | 3,317(25.3) |  | 1,184(20.0) | 1,414(25.2) | 2,598(22.6) |
| 4 (Highest) | 1,229(21.9) | 271(4.5) | 1,500(12.7) |  | 1,087(26.6) | 453(8.7) | 1,540(16.6) |  | 2,236(39.6) | 861(15.2) | 3,097(27.4) |  | 3,428(52.5) | 1,358(20.7) | 4,786(36.5) |  | 3,666(62.0) | 1,412(25.2) | 5,078(44.1) |
| ***Health service utilization*** |  |  |  |  |  |  |  |  |  |  |  |  |  |  |  |  |  |  |  |
| Inpatient service |  |  |  |  |  |  |  |  |  |  |  |  |  |  |  |  |  |  |  |
| Yes | 893(15.9) | 1,061(17.4) | 1,954(16.7) |  | 837(20.5) | 1,063(20.5) | 1,900(20.4) |  | 1,252(22.2) | 1,282(22.6) | 2,534(22.4) |  | 1,571(24.1) | 1,621(24.7) | 3,192(24.4) |  | 1,393(23.6) | 1,547(27.6) | 2,940(25.3) |
| No | 4,721(84.1) | 5,025(82.6) | 9,746(83.3) |  | 3,256(79.6) | 4,134(79.6) | 7,390(79.6) |  | 4,390(77.8) | 4,385(77.4) | 8,775(77.6) |  | 4,960(76.0) | 4,940(75.3) | 9,900(75.6) |  | 4,519(76.4) | 4,061(72.4) | 8,580(74.5) |
| Outpatient service |  |  |  |  |  |  |  |  |  |  |  |  |  |  |  |  |  |  |  |
| Yes | 1,675(29.8) | 2,332(38.3) | 4,007(34.2) |  | 1,487(36.3) | 2,174(41.8) | 3,661(39.4) |  | 2,000(35.5) | 2,483(43.8) | 4,483(39.6) |  | 2,362(36.2) | 2,884(44.0) | 5,246(59.9) |  | 2,177(36.8) | 2,646(47.2) | 4,823(41.9) |
| No | 3,939(70.2) | 3,754(61.7) | 7,693(65.8) |  | 2,606(63.7) | 3,023(58.2) | 5,629(60.6) |  | 3,642(64.6) | 3,184(56.2) | 6,826(60.4) |  | 4,169(63.9) | 3,677(56.0) | 7,846(40.1) |  | 3,735(63.2) | 2,962(52.8) | 6,697(58.1) |
| Health insurance |  |  |  |  |  |  |  |  |  |  |  |  |  |  |  |  |  |  |  |
| NRCMS | 1,707(30.4) | 4,929(81.0) | 6,636(56.7) |  | 1,627(39.8) | 4,474(86.2) | 6101(65.7) |  | 2,343(41.5) | 4,880(86.2) | 7223(63.9) |  | 2,934(44.9) | 5,683(86.6) | 8617(65.8) |  | 2,583(43.7) | 4,843(86.4) | 7426(64.5) |
| UEBMI | 1,167(20.8) | 122(2.0) | 1289(11.0) |  | 1,025(25.0) | 190(3.7) | 1215(13.1) |  | 1,547(27.4) | 225(4.0) | 1772(15.7) |  | 1,754(26.9) | 265(4.0) | 2019(15.4) |  | 1,656(28.0) | 219(3.9) | 1875(16.3) |
| URBMI | 691(12.3) | 109(1.8) | 800(6.9) |  | 507(12.4) | 69(1.3) | 576(6.2) |  | 845(15.0) | 122(2.2) | 967(8.6) |  | 890(13.6) | 111(1.7) | 1001(7.7) |  | 848(14.3) | 83(1.5) | 931(8.1) |
| SMI | 1,152(20.5) | 345(5.7) | 1497(12.8) |  | 339(8.3) | 103(2.0) | 442(4.8) |  | 403(7.2) | 98(1.7) | 5.1(4.4) |  | 321(4.9) | 67(1.0) | 388(3.0) |  | 293(5.0) | 70(1.3) | 363(3.1) |
| No health insurance | 897(16.0) | 581(9.6) | 1478(12.6) |  | 595(14.5) | 356(6.9) | 951(10.2) |  | 502(8.9) | 335(5.9) | 837(7.4) |  | 632(9.7) | 435(6.6) | 1067(8.1) |  | 532(9.0) | 393(7.0) | 925(8.0) |
| Total |  |  | 11,700(100.0) |  |  |  | 9,290(100.0) |  |  |  | 11,309(100.0) |  |  |  | 13,092(100.0) |  |  |  | 11,520(100.0) |

**Supplementary Table 2 Linear Probability Model of CHE incidence in urban households from 2010-2018**

| **Variables** | **2010** | |  | **2012** | |  | **2014** | |  | **2016** | |  | **2018** | |
| --- | --- | --- | --- | --- | --- | --- | --- | --- | --- | --- | --- | --- | --- | --- |
|  | **β** | **S.E** |  | **β** | **S.E** |  | **β** | **S.E** |  | **β** | **S.E** |  | **β** | **S.E** |
| Gender | -0.004 | 0.009 |  | 0.012 | 0.010 |  | -0.001 | 0.007 |  | 0.012 | 0.010 |  | 0.002 | 0.006 |
| Age | 0.003^***^ | 0.000 |  | 0.003^***^ | 0.000 |  | 0.002^***^ | 0.000 |  | 0.003^***^ | 0.000 |  | 0.001^***^ | 0.000 |
| Marriage | 0.009 | 0.013 |  | -0.010 | 0.014 |  | 0.005 | 0.010 |  | -0.010 | 0.014 |  | 0.002 | 0.008 |
| Education | -0.013^***^ | 0.005 |  | -0.006 | 0.005 |  | -0.008^**^ | 0.004 |  | -0.006 | 0.005 |  | -0.003 | 0.004 |
| Self-rated health | -0.122^***^ | 0.012 |  | -0.042^***^ | 0.007 |  | -0.044^***^ | 0.005 |  | -0.042^***^ | 0.007 |  | -0.034^***^ | 0.005 |
| 65 and older | 0.090^***^ | 0.012 |  | 0.056^***^ | 0.013 |  | 0.039^***^ | 0.010 |  | 0.056^***^ | 0.013 |  | 0.031^***^ | 0.009 |
| Family size | -0.047^***^ | 0.007 |  | -0.046^***^ | 0.007 |  | -0.028^***^ | 0.006 |  | -0.046^***^ | 0.007 |  | -0.038^***^ | 0.005 |
| Economic status | -0.025^***^ | 0.004 |  | -0.029^***^ | 0.004 |  | -0.018^***^ | 0.004 |  | -0.029^***^ | 0.004 |  | -0.026^***^ | 0.004 |
| Chronic disease | 0.030^***^ | 0.010 |  | 0.030^***^ | 0.011 |  | 0.011 | 0.008 |  | 0.030^***^ | 0.011 |  | 0.000 | 0.008 |
| Inpatient service | 0.185^***^ | 0.011 |  | 0.152^***^ | 0.012 |  | 0.114^***^ | 0.009 |  | 0.152^***^ | 0.012 |  | 0.102^***^ | 0.008 |
| Outpatient service | 0.025^***^ | 0.009 |  | 0.029^***^ | 0.010 |  | 0.002 | 0.008 |  | 0.029^***^ | 0.010 |  | 0.014^**^ | 0.007 |
| Insurance | -0.001 | 0.003 |  | 0.009^***^ | 0.004 |  | 0.002 | 0.003 |  | 0.009^***^ | 0.004 |  | 0.003 | 0.003 |
| Constant | 0.406 | 0.055 |  | 0.155 | 0.049 |  | 0.153 | 0.037 |  | 0.155 | 0.049 |  | 0.193 | 0.034 |
| R-squared | 0.164 | |  | 0.138 | |  | 0.110 | |  | 0.138 | |  | 0.111 | |
| F-test | 91.718 | |  | 54.532 | |  | 57.752 | |  | 54.532 | |  | 61.599 | |
| AIC | 2521.898 | |  | 1559.358 | |  | 805.015 | |  | 1559.358 | |  | -412.641 | |
| N | 5614 | |  | 4092 | |  | 5640 | |  | 4092 | |  | 5912 | |
| P | 0.000 | |  | 0.000 | |  | 0.000 | |  | 0.000 | |  | 0.000 | |
| BIC | 2608.127 | |  | 1641.477 | |  | 891.305 | |  | 1641.477 | |  | -325.739 | |

**** p<0.01, ** p<0.05, * p<0.1*

**Supplementary Table 3 Linear Probability Model of CHE incidence in rural households from 2010-2018**

| **Variables** | **2010** | |  | **2012** | |  | **2014** | |  | **2016** | |  | **2018** | |
| --- | --- | --- | --- | --- | --- | --- | --- | --- | --- | --- | --- | --- | --- | --- |
|  | **β** | **S.E** |  | **β** | **S.E** |  | **β** | **S.E** |  | **β** | **S.E** |  | **β** | **S.E** |
| Gender | -0.005 | 0.012 |  | -0.041^***^ | 0.012 |  | -0.008 | 0.009 |  | -0.002 | 0.008 |  | -0.016^*^ | 0.008 |
| Age | 0.004^***^ | 0.000 |  | 0.004^***^ | 0.000 |  | 0.003^***^ | 0.000 |  | 0.002^***^ | 0.000 |  | 0.002^***^ | 0.000 |
| Marriage | 0.011 | 0.015 |  | -0.020 | 0.016 |  | -0.016 | 0.013 |  | -0.004 | 0.011 |  | -0.006 | 0.011 |
| Education | -0.021^***^ | 0.007 |  | -0.011 | 0.007 |  | -0.005 | 0.006 |  | 0.002 | 0.005 |  | 0.000 | 0.006 |
| Self-rated health | -0.090^***^ | 0.011 |  | -0.043^***^ | 0.006 |  | -0.038^***^ | 0.006 |  | -0.041^***^ | 0.005 |  | -0.030^***^ | 0.005 |
| 65 and older | 0.034^***^ | 0.012 |  | 0.061^***^ | 0.012 |  | 0.026^**^ | 0.010 |  | 0.033^***^ | 0.009 |  | 0.031^***^ | 0.010 |
| Family size | -0.054^***^ | 0.006 |  | -0.055^***^ | 0.007 |  | -0.037^***^ | 0.006 |  | -0.045^***^ | 0.005 |  | -0.046^***^ | 0.005 |
| Economic status | -0.046^***^ | 0.005 |  | -0.029^***^ | 0.005 |  | -0.023^***^ | 0.004 |  | -0.030^***^ | 0.004 |  | -0.029^***^ | 0.004 |
| Chronic disease | 0.042^***^ | 0.011 |  | 0.037^***^ | 0.011 |  | 0.009 | 0.010 |  | 0.024^**^ | 0.009 |  | 0.021^**^ | 0.009 |
| Inpatient service | 0.206^***^ | 0.012 |  | 0.192^***^ | 0.012 |  | 0.151^***^ | 0.010 |  | 0.135^***^ | 0.010 |  | 0.132^***^ | 0.009 |
| Outpatient service | 0.018^*^ | 0.010 |  | 0.020^*^ | 0.010 |  | 0.021^**^ | 0.009 |  | -0.007 | 0.009 |  | -0.002 | 0.009 |
| Insurance | -0.006 | 0.006 |  | 0.008 | 0.006 |  | 0.008 | 0.006 |  | 0.004 | 0.006 |  | 0.003 | 0.006 |
| Constant | 0.384 | 0.060 |  | 0.224 | 0.056 |  | 0.145 | 0.047 |  | 0.200 | 0.043 |  | 0.173 | 0.045 |
| R-squared | 0.137 | |  | 0.142 | |  | 0.113 | |  | 0.115 | |  | 0.124 | |
| F-test | 80.034 | |  | 71.299 | |  | 59.817 | |  | 68.204 | |  | 66.054 | |
| AIC | 4426.148 | |  | 3637.620 | |  | 2728.422 | |  | 2910.019 | |  | 2126.814 | |
| N | 6086 | |  | 5192 | |  | 5660 | |  | 6334 | |  | 5608 | |
| P | 0.000 | |  | 0.000 | |  | 0.000 | |  | 0.000 | |  | 0.000 | |
| BIC | 4513.426 | |  | 3722.833 | |  | 2814.758 | |  | 2997.817 | |  | 2213.030 | |

**** p<0.01, ** p<0.05, * p<0.1*
